# Farrerol mitigates lipopolysaccharide-induced acute lung injury through dual regulation of oxidative stress and inflammation via Nrf2 pathway

**DOI:** 10.1515/biol-2025-1241

**Published:** 2026-02-02

**Authors:** Shun Qi, Jianbo Chang, Guoxing Li, Xianjing Zeng, Fangying Liu

**Affiliations:** Intensive Care Unit, Taizhou Traditional Chinese Medicine Hospital, Taizhou, Zhejiang, 317700, China; Department of Emergency, The First Affiliated Hospital of Chongqing Medical University, Chongqing, 400064, China; Department of Emergency, The Affiliated Dazu’s Hospital of Chongqing Medical University, Chongqing, 402360, China; Department of Outpatient, General Hospital of Western Theater of Chinese People’s Liberation Army, Chengdu, Sichuan, 610000, China; General Practice Medicine, Affiliated Hospital of Jinggangshan University, Ji’an, Jiangxi, 343000, China; Department of Critical Care Medicine, Affiliated Hospital of Jinggangshan University, Ji’an, Jiangxi, 343000, China

**Keywords:** ferrerol, lipopolysaccharide, acute lung injury, NrF2, oxidative stress, inflammation

## Abstract

Acute lung injury (ALI) is a serious illness of the respiratory system that leads to lung damage. Clinical outcomes are limits and identification of a novel drug for ALI without side effects on patients. This study investigated the effects of farrerol (FRL) in a mouse model of lipopolysaccharide (LPS)-induced ALI. Mice were subjected to FRL pre-treatment 1 h prior to LPS administration daily for 7 days. Then, lung tissue was examined in various experiments, i.e. histopathology, antioxidant status, western blot and semi-quantitative polymerase chain reaction to confirm the inflammatory response in ALI experimental models. The obtained results stated that FRL treatment alleviates LPS-mediated pathological changes, such as alveolar wall thickening, decreasing lung edema, and inflammation infiltration in the lung tissue. Moreover, LPS-induced TBARS levels were modulated by FRL treatment in mice. While enhancing antioxidant enzyme activities by FRL treatment on LPS-induced mouse models. FRL also suppressed LPS-induced expression of COX-2, iNOS, TNF-α, IL-6 and IL-β1 in mouse models. In addition, FRL has a good binding interaction; therefore, it has restored the LPS-induced Nrf2 expression. These findings indicate that FRL holds a significant therapeutic agent for ALI by offering Nrf2 mediated inhibition of oxidative stress and inflammation in mouse model.

## 1 Introduction

Acute lung injury (ALI), otherwise known as acute respiratory distress syndrome (ARDS), is characterized by a severe respiratory complaint manifest by prompt interruption of the alveolar-capillary membrane, resulting in increased inflammation, pulmonary edema, and profound hypoxemia [1]. ALI can also result in inflammation associated with several illnesses, such as pneumonia, sepsis, reperfusion injury, and acute pancreatitis. Therefore, it exhibits substantial morbidity and mortality around the globe [2]. So far, there is no effective therapy for ALI despite having advanced supportive care. Reports are documented that with best supportive care, it depends on the ventilation strategies to protect the lungs [3]. Clinical examination has confirmed that infections with gram-negative bacteria play a crucial role in the progression of ALI [4]. Lipopolysaccharide (LPS) is a major endotoxin that occurs in Gram-negative bacteria’s outer membrane cell wall, which can induce reactive oxygen species (ROS) mediated inflammation in lung epithelial cells, alveolar cells, etc. [5].

Oxidative stress arises when there is an imbalance between ROS and the body’s antioxidant defenses during the cellular damage [6]. Elevated levels of antioxidants play a vital role in neutralizing ROS, thereby safeguarding cells from oxidative harm [7]. Key antioxidant enzymes such as superoxide dismutase (SOD), catalase, and glutathione peroxidases (GPx) help to protect lung cells from the damaging effects of ROS and lipid peroxidation [8]. When cells fail to produce sufficient antioxidants, the redox balance in lung tissues becomes disrupted. This imbalance, driven by excessive ROS production, leads to the activation of inflammatory genes and the release of pro-inflammatory cytokines [9]. ALI mainly exhibits a systematic process of inflammation in the lung, and its pathogenesis is mainly known to activate alveolar macrophages and various inflammatory cells that produce tumor necrosis factor-alpha (TNF-α) and various pro-inflammatory cytokines and chemokines, such as IL-6 and IL-1β [10]. Inducible nitric oxide synthase (iNOS) and cyclooxygenase-2 (COX-2) activation lead to enhanced overproliferation of macrophages, which release other inflammatory cytokines and chemokines [11]. However, the regulation of inflammatory mediators and macrophage-associated cytokines and chemokines production was unclear.

Nuclear erythroid factor 2 (NrF2) is a potent transcriptional factor that belongs to the antioxidant protein family, which is involved in scavenging ROS and inhibiting oxidative stress and inflammation [12]. During oxidative stress in the cells, NrF2 participates in entering the nucleus from the cytosol and begins its transcriptional role of antioxidant enzymes, antioxidant genes, and ultimately diminishes oxidative damage [13]. In addition, phosphorylation of Nrf2 by kinase activity is expected to enable the release of Nrf2, resulting in inhibition of oxidative stress and inflammation [14]. Consequent activation of Nrf2 signaling is considered a novel approach to enhance antioxidant defense elements and impede pro-inflammatory cytokines production, therefore, might prevent ALI. Natural compounds or drugs directly targeting the Nrf2 signaling to prevent ALI. There are numerous phenolic agents from plants such as epigallocatechin gallate (EGCG), quercetin, curcumin, and terpenoids such as ginsenosides modulated Nrf2 signaling and improved the lung health in experimental models [15], [16], [17], [18]. Hence, NrF2 activators by the natural products might provide new tactics to intrude ALI.

Numerous treatment strategies have been followed over the last few decades to enhance the efficacy and safety of ALI patients. However, the therapeutics and treatment strategies failed to respond to conventional therapy or produced several side effects [19]. Hence, there is a need to discover a novel therapeutic drug that specifically targets oxidative stress and inflammation-mediated lung injury. Farrerol (FRL), a bioactive flavanone derived from *Rhododendron dauricum* and related species within the *Rhododendron genus*, possesses well-documented expectorant properties [20]. It exerts a direct modulatory effect on the respiratory epithelium by stimulating ciliary motility, thereby facilitating the clearance of mucus and entrapped particulates from the tracheobronchial tree through enhanced mucociliary transport mechanisms [21]. Clinically, FRL is predominantly employed in the management of chronic bronchitis, particularly in cases characterized by persistent sputum production and increased mucus viscosity [22]. Beyond its respiratory applications, FRL demonstrates potent anti-inflammatory activity in the gastrointestinal tract, largely mediated through the downregulation of the MAPK and NF-κB signaling pathways [23]. Furthermore, in the central nervous system, FRL has been shown to attenuate neuroinflammatory processes via modulation of the NRF2/KEAP1 axis, thereby suppressing microglial activation and oxidative stress [24]. However, despite this broad pharmacological spectrum, the FRL on LPS-associated oxidative stress and inflammation signaling has not yet been elucidated. Therefore, in this study, we have investigated the role of FRL in LPS-associated oxidative stress and inflammatory signaling through modulating the function of NrF2 signaling in experimental animal models.

## 2 Materials and methods

### 2.1 Animals and treatment

Pathogen-free male C57BL/6 mice, aged 7–8 weeks and weighing between 22 and 25 g, were procured from Gempharmatech Co., Ltd. The animals were acclimatized under controlled environmental conditions, maintained at a temperature of 22 ± 1 °C and relative humidity of 55 ± 5 %, with a 12-h light/dark cycle. They were provided with sterile food and water *ad libitum*. All experimental protocols were reviewed and approved by the Animal Care and Use Committee of Affiliated Hospital of Jinggangshan University, Jiangxi and conducted in accordance with the Institutional Animal Ethical Committee (IAEC) guidelines for the care of experimental animals. The mice were randomly assigned to four experimental groups, with six animals per group:Group I: Normal controlGroup II: LPS (10 mg/kg body weight)Group III: FRL (40 mg/kg body weight) + LPS (10 mg/kg body weight)Group IV: FRL (50 mg/kg body weight) + LPS (10 mg/kg body weight)Mice in the untreated control group received intraperitoneal (i.p.) injections of normal saline once daily for seven consecutive days. LPS was dissolved in 0.1 % DMSO and administered intraperitoneally for seven days. FRL pre-treatment (40 and 50 mg/kg b.w.) was delivered via i.p. injection 1 h prior to LPS administration, repeated daily for the same duration. Successful establishment of the LPS-induced acute lung injury (ALI) model was confirmed by the presence of clinical symptoms such as decreased food intake, weight loss, a dull coat, lethargy, and increased respiratory rate. At the end of the treatment period, the mice were anesthetized with 1 % pentobarbital and subsequently euthanized in a humane manner. Lung tissues and bronchoalveolar lavage fluid (BALF) were harvested for further analysis.

**Ethical approval:** The research related to animal use has been complied with all the relevant national regulations and institutional policies for the care and use of animals, and has been approved by the Animal Care and Use Committee of Affiliated Hospital of Jinggangshan University, Jiangxi (Approval No. 2023-23).

### 2.2 Lung wet-to-dry weight ratio

Following euthanasia, a portion of lung tissue was promptly excised, and surface moisture was gently blotted using sterile absorbent paper to obtain the wet weight. The tissue was then placed in a drying oven at 80 °C for 24 h to determine its dry weight. The ratio of wet-to-dry (W/D) weight was subsequently calculated as an index to assess the severity of pulmonary edema [25].

### 2.3 Histological study

Following euthanasia, lung tissues were promptly harvested to assess pulmonary injury through histopathological analysis. Specimens from each experimental group were fixed in 4 % paraformaldehyde at 37 °C for 24 h. Subsequently, the tissues were sectioned into 5 μm-thick slices and stained with hematoxylin and eosin (H&E) to evaluate morphological alterations and pathological changes in lung architecture [26].

### 2.4 Determination of antioxidants and lipid peroxidations

The oxidative stress marker enzymes, such as CAT, SOD, and GPx, and TBARS, are estimated by commercial assay kits in accordance with the manufacturer’s instructions.

### 2.5 Western blotting assay

Following euthanasia, lung tissues were promptly harvested to assess protein extraction by the addition of RIPA buffer with protease cocktail inhibitor, correspondingly. After lung tissue homogenization, the protein concentration was quantified by using the Bradford reagent assay kit with the company’s instructions. The protein samples from the experimental groups were subjected to SDS-PAGE to separate the proteins present in the samples. After separation of proteins in the gel, the proteins were transferred to polyvinylidene difluoride (PVDF) membranes (Biorad) by using a wet blot system [27]. Then, the PVDF membrane was blocked with 5 % blocking reagent, which contains BSA, for 1 h. Every step between the membranes was rinsed with TBST solution. The appropriate primary antibodies were used to add the membrane for 6 h, followed by adding secondary antibodies for 45 min. After incubation, the expression of bands in the membrane was developed by the addition of chemiluminescent (ECL) and scanned by a gel documentation system (Azure, Austria).

### 2.6 Semi-quantitative polymerase chain reaction

Following euthanasia, lung tissues from various experimental groups were collected and subjected to total RNA extraction using TRIzol reagent (Invitrogen, USA). Next, the conversion of cDNA from whole RNA was performed using the reverse transcriptase kit (Invitrogen, Life Technologies). Semi-Quantitative PCR reactions were accomplished by adding 2 μL cDNA and 10 μL amplicon Master Mix (BioRad), 4 μL of forward primer, and 4 μL of reverse primer in a Thermocycler Detection system (Applied Biosystems). The expression levels of pointing mRNAs were normalized to those of the housekeeping gene GAPDH [28].

### 2.7 Molecular docking

The PyRx (0.8) tool was used to evaluate the binding affinity of FRL with the NrF2 protein interaction. The NMR-based crystal structure of the NrF2 protein was obtained from the RCSB Protein Data Bank, and its PDB ID is 7O7B. The 3D conformer SDF file of ligand farrerol (FRL) was downloaded from the PubChem database. The docking study was carried out using PyRx software the detailed protocol was followed by Wei et al. 2024 [29].

### 2.8 Statistical analysis

All the presented data are mean ± standard deviation (SD) from at least three independent experiments. *N* = 6 in each experimental group. Data are statistically employed by using SPSS software (Version 24.0, USA), and one-way analysis of variance enables various comparisons. A *P*-value < 0.05 was deemed statistically significant.

## 3 Results

### 3.1 FRL treatment alleviates LPS-induced acute lung injury in C57BL/6 mouse models

**Figure 1:**
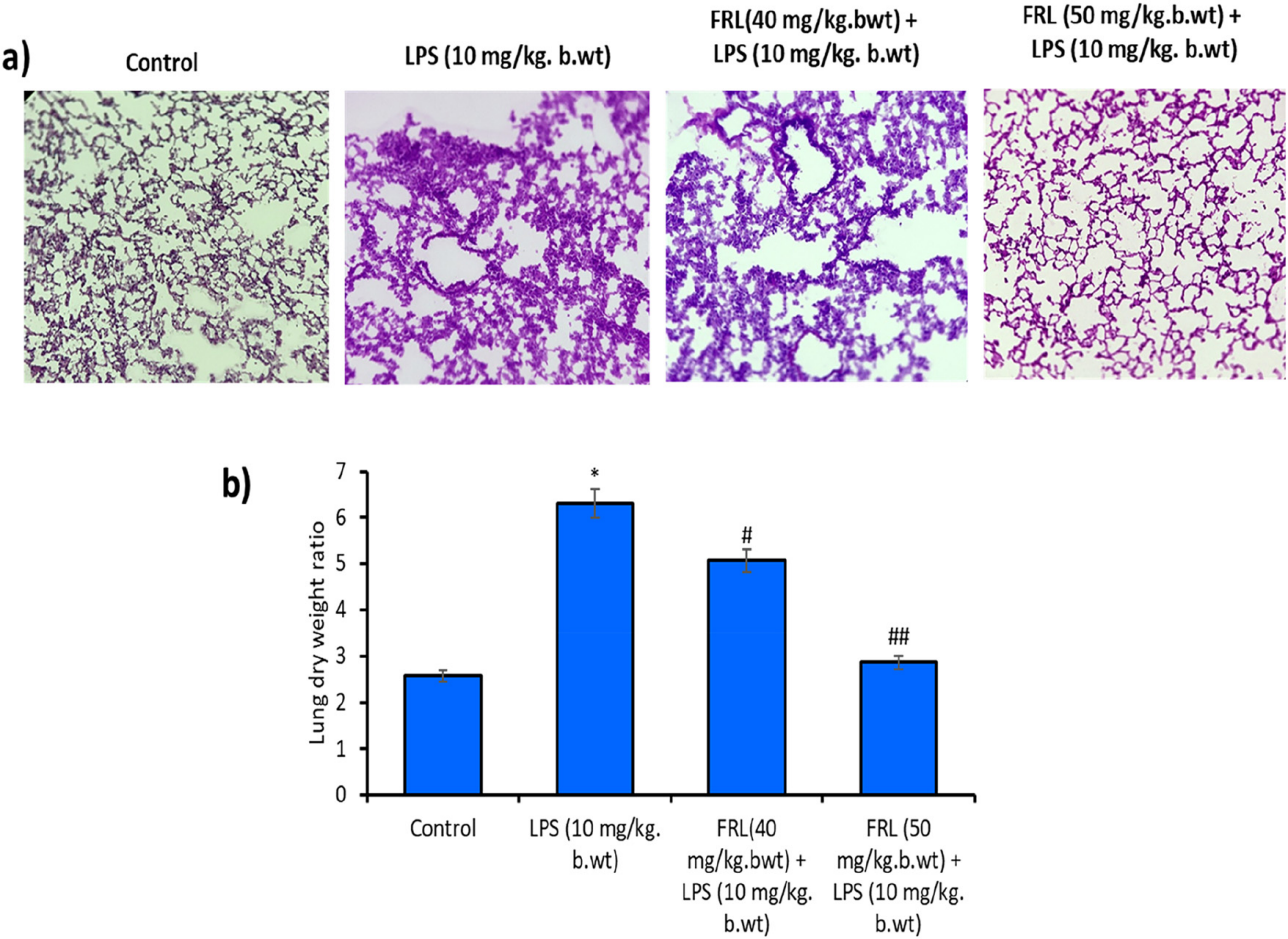
FRL on LPS exposed acute lung injury (ALI) in C57BL/6 mouse models. (a) Histopathological examination of FRL and/or LPS treatment-mediated ALI was studied by eosin and hematoxylin staining. The representative image was given 40× and taken by a bright-field microscope (Nikon, Japan). (b) Bar diagram represents the FRL and/or LPS treatment-mediated lung dry weight ratio. All the presented data are mean ± SD from at least three independent experiments. *N* = 6 in each group. *P < 0.05 versus NC group. #*P* < 0.05 and ##*P* < 0.01 versus LPS group.

A histopathological study was performed to evaluate the effect of FRL on LPS-mediated acute lung injury in the mouse model using Hematoxylin and Eosin (H&E) staining. The H&E-stained lung tissue sections reveal that LPS significantly resulted in the pathologic alterations of the lung tissue through increasing inflammation compared to the Control group (Figure 1a). However, FRL treatment alleviates the inflammation caused by LPS in animal models. Moreover, alveolar wall thickening was observed in the LPS group, and it was reversed by FRL treatment.

In addition, lung inflammation and edema were evaluated by lung dry weight in the animal models. Figure 1b shows that the increased lung dry weight in the LPS group is greater than that of the control. However, a significant decrease in lung dry weight in FRL treated LPS-induced mouse models. These results revealed that FRL protects against LPS-induced in models.

### 3.2 Effect of FRL on LPS-exposed lipid peroxidation and antioxidants in C57BL/6 mouse models

**Figure 2:**
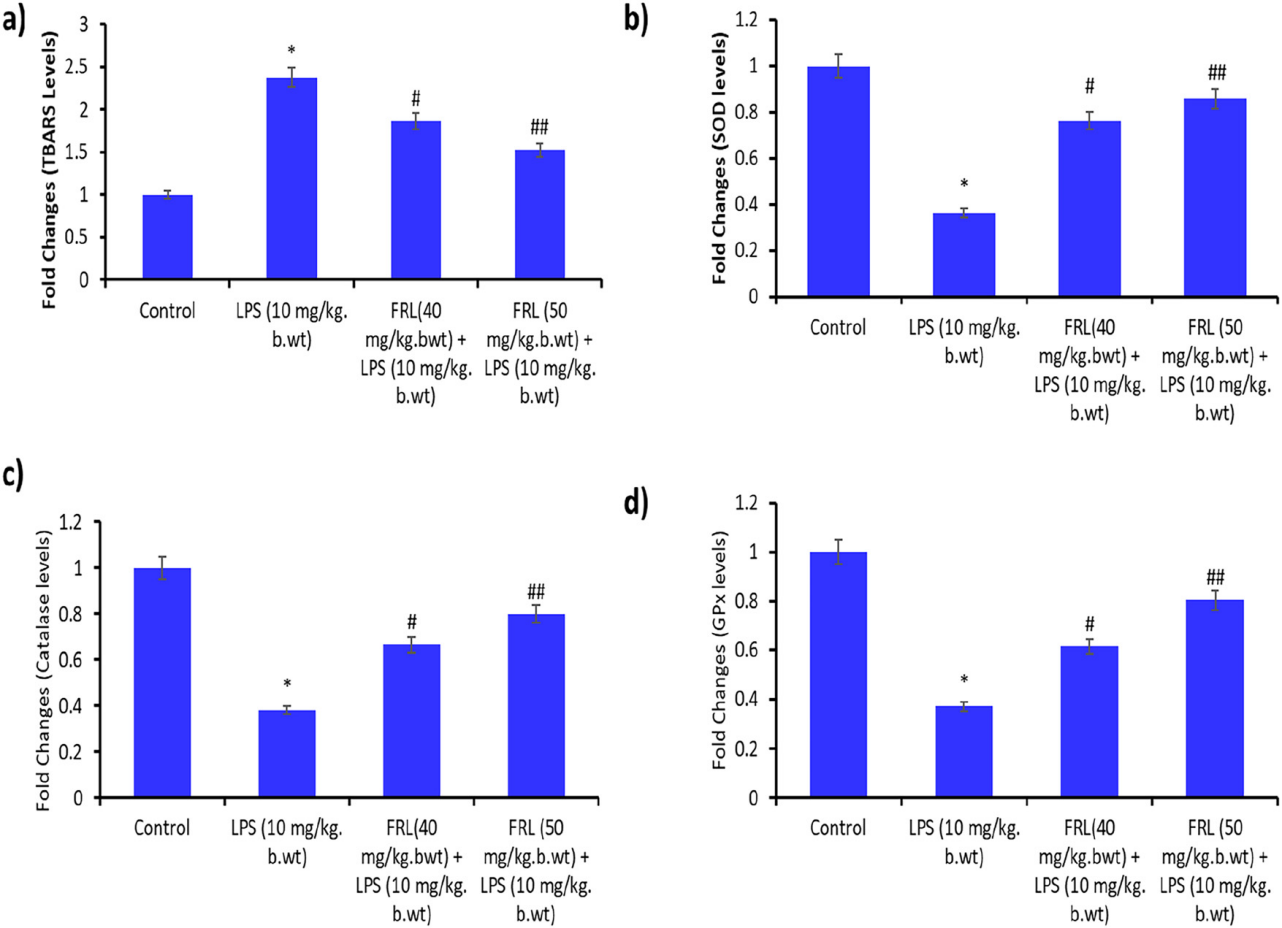
FRL on LPS exposed lipid peroxidation and antioxidants in C57BL/6 mouse models. (a) TBARS levels. SOD levels were expressed in units/mg protein. (c) Catalase levels expressed in units/mg protein. (d) GPx expressed in units/mg protein. All the presented data are mean ± SD from at least three independent experiments. *P < 0.05 versus NC group. #*P* < 0.05 and ##*P* < 0.01 versus LPS group.

The Thiobarbituric Acid Reactive Substance (TBARS) assay is commonly used to measure the oxidative damage to lipids through the lipid peroxidation process. Higher TBARS levels in the LPS group than in the control group indicate increased oxidative damage. The FRL treatment (40 mg/kg bwt and 50 mg/kg bwt) reversed the oxidative damage caused by LPS (Figure 2a).

Superoxide Dismutase (SOD), catalase, and Glutathione Peroxidase (GPX) are significant antioxidant enzymes in protecting the cells from oxidative stress [30]. The decreased SOD, catalase, and GPX levels in the LPS group compared to the control indicate oxidative stress, and further FRL treatment restored the SOD, catalase, and GPX levels, thus minimizing the oxidative stress induction by LPS (Figure 2b–d). These results suggest that FRL prevents TBARS and depletion of antioxidants, thereby preventing LPS-induced oxidative stress in mouse models.

### 3.3 Effect of FRL modulates LPS-induced Nrf2 signaling in mouse models

**Figure 3:**
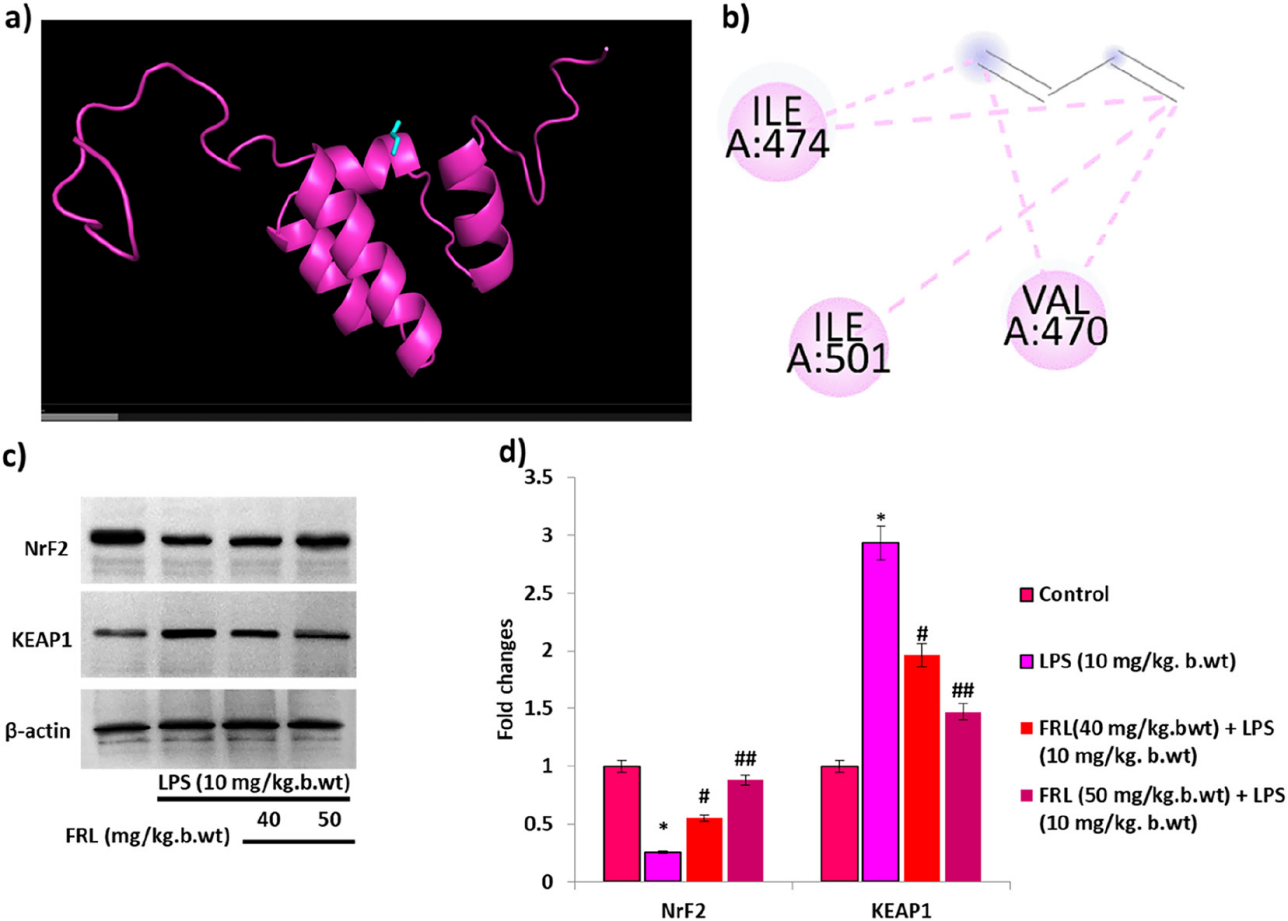
FRL on LPS induced NrF2 signaling in mouse models. (a) 3D structure of the binding interaction of NrF2 with FRL analyzed by Autodock. (b) 2D structure of interaction between the amino acid sequence in the NrF2 protein against FRL. (c) Western blot analysis of NrF2 and KEAP1 protein expression in LPS and/or FRL exposed mouse models. (d) Bar diagram represents the fold changes of NrF2 and KEAP1 studied by densitometric analysis using Image J software. β-actin is considered an internal control, confirming equal loading protein concentrations in the samples. All the presented data are mean ± SD from at least three independent experiments. *P < 0.05 versus NC group. #*P* < 0.05 and ##*P* < 0.01 versus LPS group.

NrF2 is a major antioxidant protein, and whether FRL enhances the expression of NrF2 in LPS-induced mouse models. Firstly, *In silico* investigation reveals that the molecular interaction between the FRL and Nrf2 was evaluated by Molecular docking. It shows FRL has a strong binding interaction with the Nrf2 protein, with a binding affinity of −8.8 kcal/mol. The 3-D and 2-D interaction images of Nrf2 and FRL complex depict the interaction of amino acid residues ILE A:474, ILE A:501, and VAL A:470 (Figure 3a and b).

Nrf2 and Kelch-like ECH-associated protein 1 (KEAP1) play a critical role in acute lung injury by regulating the antioxidant enzymes [31]. Hence, western blot analysis was performed to evaluate the protein expression of KEAP1 and Nrf2 in the LPS and FRL treated LPS-induced mouse models. The results show that LPS exposure to the mouse models notably downregulated Nrf2 protein expression in the lung tissue compared to the untreated control. However, Nrf2 protein expression is restored by FRL treatment in LPS-induced mouse models. Moreover, LPS exposure has significantly upregulated KEAP1 protein expression in the lung tissue compared to untreated controls, and FRL alleviates its protein expression in LPS-induced mouse models (Figure 3c). FRL treatment enhances Nrf2 protein expression while reducing KEAP1 protein expression, thereby significantly alleviating oxidative stress in LPS-induced mouse models via the Nrf2 pathway.

### 3.4 FRL suppresses the inflammatory protein expression in LPS-induced mouse models

**Figure 4:**
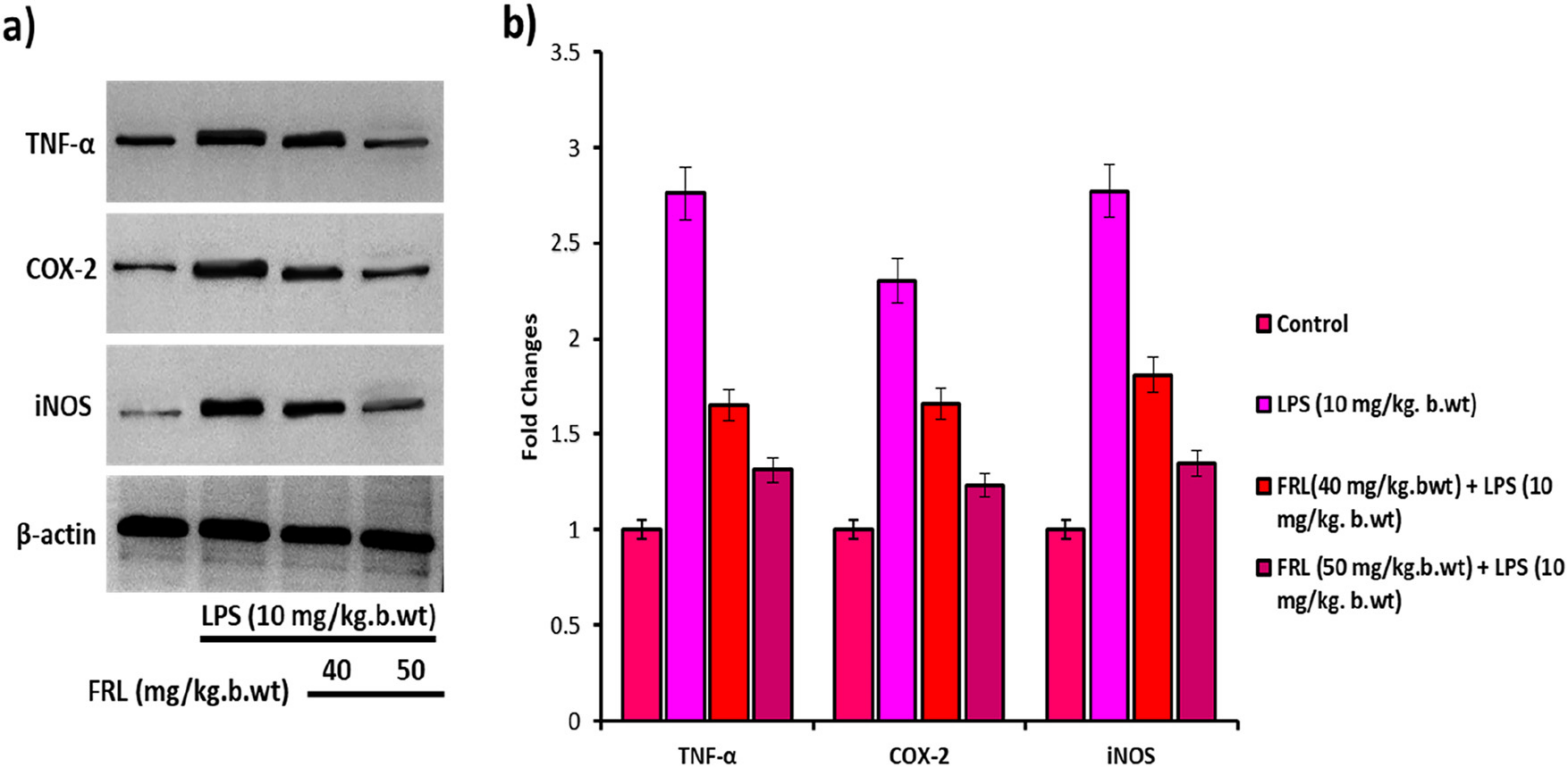
FRL on LPS induced inflammatory protein expressions in mouse models. (a) Western blot analysis of TNF-α, COX-2, and iNOS protein expression in LPS and/or FRL exposed mouse models. (b) Bar diagram represents the fold changes of TNF-α, COX-2, and iNOS studied by densitometric analysis using Image J software. β-actin is considered an internal control, confirming equal loading protein concentrations in the samples. All the presented data are mean ± SD from at least three independent experiments. *P < 0.05 versus NC group. #*P* < 0.05 and ##*P* < 0.01 versus LPS group.

The inflammatory proteins, such as TNF-α, COX-2, and iNOS, play a significant role in mediating the initiation and progression of inflammation in ALI [32]. The Western blot image shows that LPS exposure to the mouse model increases the protein expression of TNF-α, COX-2, and iNOS in the lung tissue when compared to the untreated control. However, FRL treatment effectively decreased the protein expression of TNF-α, COX-2, and iNOS in LPS induction in mouse models (Figure 4). Thereby, FLP treatment downregulates the protein expression and minimizes inflammation in LPS-induced mouse models.

### 3.5 FRL reverses the Inflammatory cytokines mRNA expression in LPS-induced Mouse models

**Figure 5:**
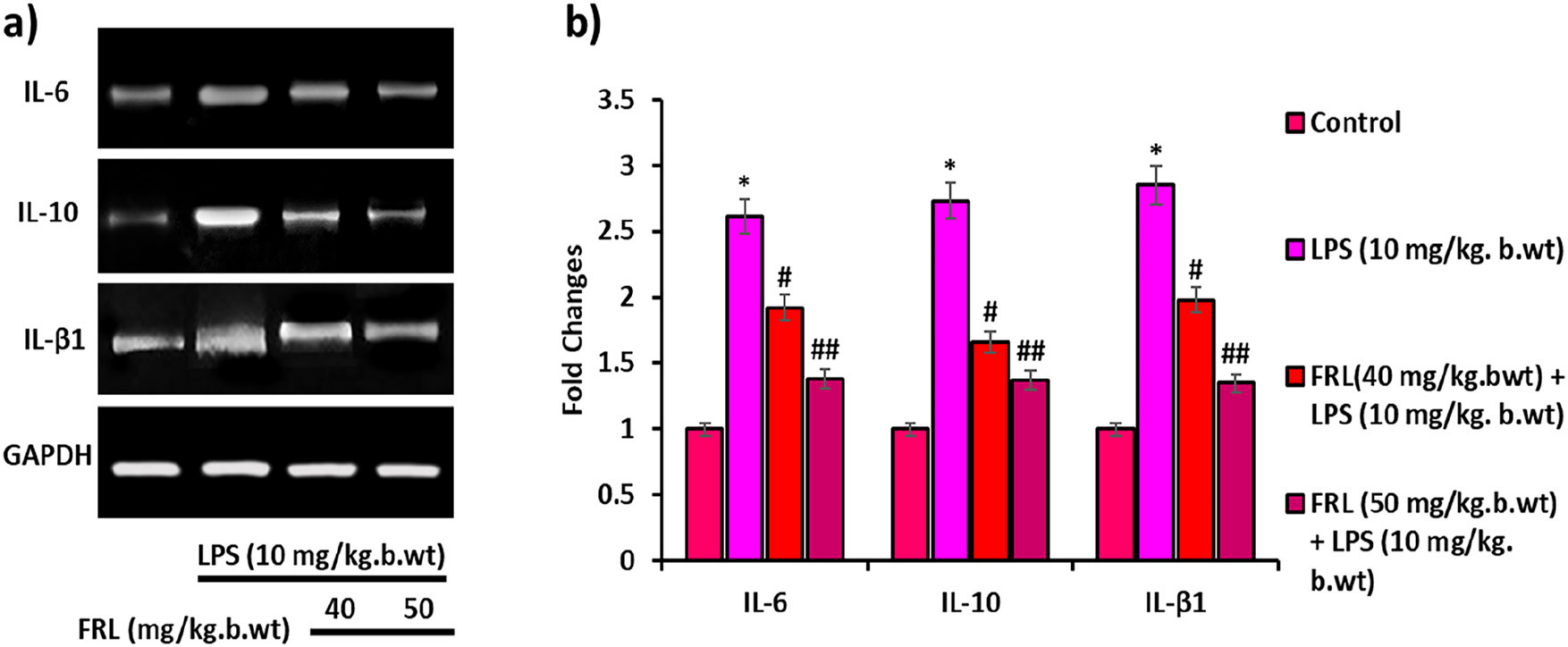
FRL on LPS induced inflammatory cytokines mRNA expressions in mouse models. (a) PCR analysis of IL-6, IL-10, and IL-β1 mRNA expression in LPS and/or FRL exposed mouse models. (b) Bar diagram represents the fold changes of IL-6, IL-10, and IL-β1 studied by densitometric analysis using Image J software. GAPDH is considered an internal control, confirming equal loading of mRNA concentrations in the samples. All the presented data are mean ± SD from at least three independent experiments. *P < 0.05 versus NC group. #*P* < 0.05 and ##*P* < 0.01 versus LPS group.

The semi-quantitative PCR analysis was performed to evaluate mRNA expression of inflammatory cytokines (IL-6, IL-10, and IL-β1). The Agarose gel electrophoresis (Figure 5a) reveals an amplification of IL-6, IL-10, and IL-β1 genes in LPS and FRL-LPS exposed mouse models. It shows that LPS promotes IL-6, IL-10, and IL-β1 mRNA expression in lung tissue when compared to untreated controls. However, its mRNA expression was significantly downregulated by subsequent FRL treatment followed by LPS exposure in lung tissue. The band intensity corresponds to the amplification of the gene bands, and its quantification was measured by ImageJ software (Figure 5b). The maximum activity was observed in the FRL (50 mg/kg bwt) treatment in the LPS-induced mouse model. Hence, FRL treatment significantly reduces the LPS-induced inflammatory responses by inhibiting the inflammatory cytokines, thereby preventing ALI in experimental mouse models.

## 4 Discussion

This study investigated the role of farrerol (FRL) treatment in LPS-exposed inflammatory responses in experimental mouse models, specifically focused on inflammatory mediators, oxidative stress, and Nrf2 signaling. Acute lung inflammation is a major issue for respiratory-related diseases and has received significant attention in the development of novel drugs [33]. Presently, research on ALI treatment emphasizes natural-based anti-inflammatory drugs due to their antioxidant potential and no side effects [34]. FRL is considered to have the strongest antioxidant properties and effectively scavenges free radicals and inflammatory responses in various organs [35]. Inflammation is a major process that is implicated in the progression of lung damage or injury. It has been activated by the production of numerous cytokines and chemokines, which coordinate to cause severe lung injury [36]. Lipopolysaccharides (LPS) are endotoxins secreted by gram-negative bacteria that produce ROS-mediated inflammatory reactions [37]. In this study, mice were exposed to LPS daily once for seven consecutive days, showing severe edema, inflammatory cells, and pathological features, which were confirmed by histopathological analysis. These changes were reverted by the mice treated with FRL following LPS exposure. These results stated that FRL is a promising candidate to prevent LPS-mediated ALI.

Oxidative stress is a major cellular event that promotes the inflammatory responses in the lung [38]. Lung cells are frequently exposed to oxidants, through either endogenous sources or exogenous factors, which leads to lung injury [39]. Several studies have substantially reported that oxidative stress plays an imperative role in the pathogenesis of numerous lung diseases, such as asthma, pulmonary fibrosis, ALI, and lung cancer [40]. In this study, TBARS levels were reduced by the FRL treatment following LPS-induced mouse models. In addition, the suppression of SOD, catalase, and GPX levels in the LPS group compared to the control indicates oxidative stress, and further FRL treatment restored the SOD, catalase, and GPX levels, thus minimizing the oxidative stress induction by LPS. Previously, red ginseng polysaccharides have been shown to prevent ALI through modulating the function of oxidative stress [41]. Moreover, farrerol also reported that to mitigates cisplatin-induced acute kidney injury by reducing levels of lipid peroxidation markers and enhancing antioxidants such as SOD and GSH [42]. Based on these results, we also confirmed that FRL prevents oxidative stress during LPS exposure in the lung tissues.

The Nrf2 activation was directly implicated in regulating cellular redox homeostasis and oxidative stress [43]. FRL has been reported to have antioxidant properties that can efficiently constrain oxidative stress. However, the exact molecular mechanism of FRL and the oxidative stress process is still unclear. Previously, FRL treatment enhanced the major antioxidant transcriptional factor, Nrf2, to mitigate oxidative stress and inflammation in 264.7 cells [44]. Therefore, we further focused on whether FRL enhances LPS-induced Nrf2 signaling in ALI models. In our study, we confirmed that FRL has a good binding interaction with the NrF2 protein; therefore, NrF2 was enhanced and KEAP1 was downregulated by the FRL treatment in LPS-exposed animal models. Previous studies have also supported our findings, indicating that farrerol activates the Nrf2 signaling pathway, which helps protect RAW 264.7 cells from oxidative damage [44].

Inflammatory cell infiltration is a hallmark event for ALI; specifically, immune cells can contribute to the production of inflammatory cytokines and chemokines for the entire process [45]. TNF-α, IL-1β, IL-10, and IL-6 have been produced from macrophages during oxidative stress and inflammatory reactions, and these markers have been proven to be potent biomarkers for inflammation [46]. Also, COX-2 and iNOS are major proteins which is involved in the progression of inflammation in the lung [47]. In this study, we found that FRL treatment efficiently suppressed the LPS-induced expression of TNF-α, IL-1β, IL-10, IL-6, COX-2, and iNOS in mice, including mitigating ALI pathological changes of lung tissues. Oridonin, a natural phytochemical, prevents or suppresses LPS-induced ALI by restraining Nrf2-associated oxidative stress, cytokine overproduction, and NF-κB pathways [48]. The limitations of this study are primarily related to its focus on the Nrf2 pathway. However, acute lung injury (ALI) involves multiple signaling cascades, including NF-κB, MAPK, and PI3K/AKT pathways. The potential cross-talk between these pathways and Nrf2 under FRL treatment remains unexplored. Furthermore, the pharmacokinetic characteristics, including bioavailability and possible systemic toxicity of FRL, were not assessed. These factors are considered limitations of the study and warrant further investigation to support the translation of preclinical findings into clinical applications.

## 5 Conclusions

This study concluded that FRL is an antioxidant based on natural phytochemicals, which prevents the LPS-induced ALI in experimental mouse models. FRL administration tremendously inhibits inflammatory cytokines and proteins, and oxidative stress through enhancing the Nrf2 signaling in LPS-exposed mice. Hence, FRL may be used as a promising natural-based drug for treating acute lung injury without causing any side effects.
